# 快速康复外科在肺癌手术患者中应用效果的*meta*分析

**DOI:** 10.3779/j.issn.1009-3419.2016.12.05

**Published:** 2016-12-20

**Authors:** 燕 夏, 淑文 常, 敬霆 叶, 谨 薛, 余声 束

**Affiliations:** 1 225001 扬州，扬州大学临床医学院 Clinical Medical College of Yangzhou University, Yangzhou 225001, China; 2 225001 扬州，扬州市苏北人民医院胸心外科 Subei People's Hospital, Yangzhou 225001, China

**Keywords:** 肺肿瘤, 快速康复外科, 肺叶切除, 胸腔镜手术, *Meta*分析, Lung neoplasms, Fast track surgery, Lobectomy, Video-assisted thoracoscopic surgery, *Meta*-analysis

## Abstract

**背景与目的:**

快速康复外科(fast track surgery, FTS)能促进患者加速康复并缩短住院时间，并已在其他一些外科疾病中得以有效运用，但在我国肺癌手术患者中实施的安全性和有效性尚未明确。本研究采用*meta*分析的方法探讨FTS在我国肺癌手术患者中的应用效果。

**方法:**

通过国内外相关数据库检索所有符合检索条件的文献，末次检索日期为2016年1月31日，根据纳入与排除标准进行进一步筛选，采用RevMan 5.3对数据进行*meta*分析。

**结果:**

8项随机对照试验(randomized controlled trial, RCT)和5项临床对照试验(clinical controlled trial, CCT)纳入研究，共计1, 241例患者。*Meta*分析结果显示：与对照组比较，FTS组术后住院时间(MD=-3.61, 95%CI: -5.05--2.16, *P* < 0.000, 01)和胸管留置时间(MD=-2.62, 95%CI: -3.07--2.17, *P* < 0.000, 01)明显缩短、术后并发症发生率(OR=0.30, 95%CI: 0.19-0.47, *P* < 0.000, 01)和住院费用(MD=-0.92, 95%CI: -1.19--0.65, *P* < 0.000, 01)明显降低。

**结论:**

FTS能安全有效地促进我国肺癌患者快速康复，具有临床应用价值。

## Abstract (en)

**Background and objective:**

Fast track surgery (FTS) can accelerate rehabilitation and reduce postoperative hospital stay. It has been effectively applied to several surgical diseases. However, the safety and effectiveness of FTS for patients with lung cancer in China is unclear. This meta-analysis aimed to evaluate the effect of FTS undergoing lung cancer surgery in China.

**Methods:**

Using home and abroad databases to search all documents required. The deadline of retrieval was January 31, 2016. Then the studies were screened according to the inclusion and exclusion criteria. Data were analyzed by RevMan 5.3 software.

**Results:**

8 randomized controlled trials (RCTs) and 5 clinical controlled trials (CCTs) with 1, 241 patients were eligible for analysis. Compared with control group, FTS group can significantly shorten postoperative hospital time (MD=-3.61, 95%CI: -5.05--2.16, *P* < 0.000, 01) and chest tube duration (MD=-2.62, 95%CI: -3.07-2.17, *P* < 0.000, 01), reduce incidence of postoperative complications (OR=0.30, 95%CI: 0.19-0.47, *P* < 0.000, 01) and hospitalization costs (MD=-0.92, 95%CI: -1.19--0.65, *P* < 0.000, 01).

**Conclusions:**

FTS can safely and effectively accelerate the recovery of patients with lung cancer in China, it exhibits important clinical values.

肺癌是目前全球发病率与病死率最高的肿瘤^[1]^。2015年国家癌症中心数据显示，我国肺癌在2006年-2011年这5年患病率已达130.2(1/10万)，严重危害我国居民健康与生命^[2]^。快速康复外科(fast track surgery, FTS)又称加速康复外科(enhanced recovery after surgery, ERAS)，由丹麦医生Kehlet等^[3]^在2001年首次提出，指在围手术期运用已被循证医学证实的优化措施以减少手术应激反应和术后并发症，从而缩短住院时间、促进患者快速康复；其措施主要包含患者健康教育、微创手术、最佳疼痛控制、早期下床活动和肠道营养等^[4]^。FTS应用于泌尿外科、骨科、妇科等诸多外科领域中的安全性与有效性已得到证实，其中以结肠手术最为成功^[5]^。但是随着临床应用的不断推广，FTS的安全问题也逐渐显露^[6]^。本研究采用*meta*分析系统评价FTS在我国肺癌手术患者中的应用效果，为其在临床推广实施提供可靠的循证医学证据。

## 1 资料与方法

### 1.1 纳入标准

#### 1.1.1 研究类型

在肺癌围手术期应用FTS的随机对照试验(randomized controlled trial, RCT)或临床对照试验(clinical controlled trial, CCT)，样本大小不限。

#### 1.1.2 研究对象

确诊为肺癌且肿瘤无侵犯其他器官、无手术禁忌症并在我国行肺癌手术的患者，不限性别、年龄。

#### 1.1.3 干预措施

实验组在围手术期应用FTS措施，对照组采用传统手术外科(conservative treatment surgery, CTS)措施。

#### 1.1.4 评价指标

术后住院时间、胸管留置时间、术后并发症发生率、术后肺部感染发生率、住院费用，凡有任意一个上述指标均可纳入本研究。

### 1.2 排除标准

凡有以下之一则排除该研究：①综述、单一队列、病例报告等非对照性研究文献；②重复发表的文献；③合并其他并发症的肺癌患者；④术前放化疗患者。

### 1.3 检索策略

以“fast track”、“FTS”、“enhanced recovery”、“ERAS program”、“accelerated rehabilitation surgery”、“multimodal perioperative care”、“multimodal rehabilitation”、“lung neoplasms”、“lung cancer”、“lung carcinoma”、“lung tumor”、“lobectomy”、“bilobectomy”、“pulmonary resection”、“radical operation”、“thoracotomy”、“thoracoscopy”、“video-assisted thoracoscopic surgery”、“快速康复”、“加速康复”、“肺癌”、“肺肿瘤”、“肺切除术”、“肺？切除”(‘？’为单字通配符)、“根治术”、“胸腔镜”等为检索词，检索Cochrane Library、PubMed、EMBASE、Web of Sciences、中国生物医学文献数据库(CBM)、中国知网(CNKI)、维普(VIP)、万方数据资源系统等，采取主题词与自由词相结合的方式，检索各库时根据各库特点相应调整，机检为主，手工检索为辅，检索时限均为从建库至2016年1月31日，同时对参考文献进行追溯，并注意会议论文、未发表的学位论文等“灰色文献”。

### 1.4 文献筛选和资料提取

由2名研究人员独立筛选文献、提取资料并进行交叉核对，如遇分歧则进行讨论或与第3名研究人员讨论决定。提取资料包括：①一般资料：题目、作者、发表日期、研究地区等；②研究特征：研究对象人口学资料、各组患者基线可比性和干预措施等；③结局指标：即评价指标。若有资料缺失或疑问，则通过邮件或电话与作者取得联系加以补充。

### 1.5 质量评价

RCT采用Cochrane协作网制定的新“偏倚风险评估”工具^[7]^进行评价；CCT采用“Newcastle-Ottawa Scale(NOS)”^[7]^进行评价，NOS的总分为9分。

### 1.6 统计学分析

采用RevMan 5.3软件对资料进行meta分析。二分类资料采用比值比(odds ratio, OR)作为合并统计量；连续资料采用均数差(mean difference, MD)作为合并统计量；所有统计量计算95%可信区间(confidence interval, CI)。各研究结果间的异质性采用χ^2^检验。若各研究间有同质性(*P*≥0.05, *I*^2^≤50%)，则选用固定效应模型(fixed effect model)；若各研究间有异质性(*P* < 0.05, *I*^2^ > 50%)，则选用随机效应模型(random effect model)。对研究设计类型(RCT和CCT)进行亚组分析，防止因研究设计类型不同对结果的产生影响。

### 1.7 发表偏倚评价

由于每一个比较纳入的RCT或CCT研究数目均少于9个，因此本研究不进行漏斗图的绘制。

## 2 结果

### 2.1 检索结果

依照检索策略初筛文献72篇；阅读题目与摘要，初步纳入文献48篇；精读全文剔除重复发表及不符合纳入标准的文献后最终纳入文献13篇^[8-20]^，均为中文文献，其中RCT 8篇、CCT 5篇(图 1)。纳入研究的病例总数为1, 241例，其中FTS组620例，CTS组621例，各组数据如年龄、性别、临床分期等均具有可比性，差异无统计学意义(*P* > 0.05)。纳入文献的基线资料见表 1。

**1:**
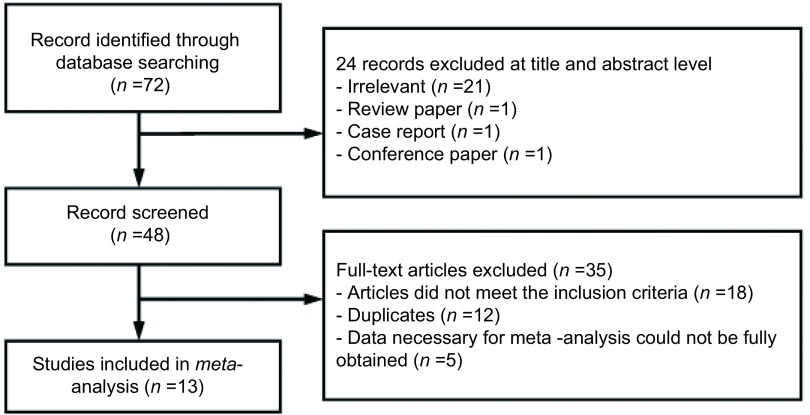
文献筛选流程图 Flow chart for study selection

**1:** 纳入文献的一般资料 The characteristics of included studies

Included study	Study type	Region	Cases			Male/female			Age (Years)			pTNM staging (Ⅰ/Ⅱ/Ⅲ)	
			FTS	CTS		FTS	CTS		FTS	CTS		FTS	CTS
Fang ZM 2015^[8]^	CCT	Guangdong	20	22		11/9	10/12		55.1±8.9	57.2±7.6		6/13/1	6/12/4
Wang JF 2015^[16]^	RCT	Jiangsu	54	54		37/17	35/19		71.4 ±6.4	70.5±5.3		24/29/1	24/29/1
Zhang XG 2015^[18]^	CCT	Guangdong	40	40		24/16	26/14		50.0±3.0	52.0±4.0		7/23/10	8/20/12
Tan NX 2014^[13]^	RCT	Hunan	30	30		19/11	18/12		56.8±6.1	57.2±5.6		6/15/9	7/13/10
Zhang C 2013^[17]^	RCT	Zhejiang	28	29		13/15	18/11		62.0±10.7	65.1±8.3		23/2/3	20/4/5
Liu YY 2015^[11]^	CCT	Fujian	83	71		44/39	37/34		57.3±11.4	58.2±12.7		—	—
Qiao K 2013^[12]^	CCT	Shenzhen	89	82		39/50	38/44		57.4 ±13.3	59.4 ± 11.8		20/64/5	19/58/5
Zhou WD 2013^[20]^	RCT	Jiangxi	51	51		27/24	23/28		70.1±5.2	71.0±5.3		16/25/10	18/21/12
Wang JY 2011^[14]^	RCT	Shanghai	36	38		25/15	22/18		62.5±4.8	64.2±7.8		5/23/12	6/20/14
Li LM 2013^[9]^	RCT	Henan	23	34		14/9	21/13		55.3±4.5	57.1±5.3		5/10/8	7 /16/12
Wang CY 2015^[15]^	CCT	Hubei	80	80		38/42	37/43		50.4±5.6	50.5±5.6		—	—
Li M 2010^[10]^	RCT	Guangxi	48	54		45/3	52/2		62.8±9.8	61.2±10.4		—	—
Zhao GQ 2010^[19]^	RCT	Yunnan	38	36		24/14	25/11		53.2±8.8	55.3±7.8		5/20/13	4/21/11
RCT: randomized controlled trial; CCT: clinical controlled trial.													

### 2.2 纳入文献的质量评价

#### 2.2.1 RCT文献质量评价

纳入8项^[9, 10, 13, 14, 16, 17, 19, 20]^RCT中，8项研究均提到随机分组，其中2项^[16, 20]^由随机数字表法产生，1项^[10]^由随机卡产生，1项^[13]^由完全随机分组法产生，其余均未提及具体方法；8项研究均未提到分配隐藏；2项^[16, 20]^研究对研究对象实施盲法，但未对结果评价者实施盲法，其余均未提及；1项研究^[14]^结果数据不完整，其余均完整；8项研究均无出现选择性报告(表 2)。

**2:** RCT文献的质量评价 Quality assessment of RCT studies

Included study	Randomized method	Allocation concealment	Blinding	Incompleteness of data	Selective outcome reporting	Other sources of bias
Wang JF 2015^[16]^	Yes	Unclear	Single-blinded	Yes	Unclear	Unclear
Tan NX 2014^[13]^	Yes	Unclear	Unclear	Yes	Unclear	Unclear
Zhang C 2013^[17]^	Unclear	Unclear	Unclear	Yes	Unclear	Unclear
Zhou WD 2013^[20]^	Yes	Unclear	Single-blinded	Yes	Unclear	Unclear
Wang JY 2011^[14]^	Unclear	Unclear	Unclear	No	Unclear	Unclear
Li LM 2013^[9]^	Unclear	Unclear	Unclear	Yes	Unclear	Unclear
Li M 2010^[10]^	Yes	Unclear	Unclear	Yes	Unclear	Unclear
Zhao GQ 2010^[19]^	Unclear	Unclear	Unclear	Yes	Unclear	Unclear

#### 2.2.2 CCT文献质量评价

纳入5项^[8, 11, 12, 15, 18]^CCT中，5项研究的研究对象选择均恰当，有详细的纳入和排除标准；5项研究的FTS组和CTS组均具有组间可比性，并控制了最重要的混杂因素，其中4项研究^[8, 11, 12, 15]^控制了其他混杂因素；5项研究均报道了结局指标和评价标准，其中1项研究^[12]^描述了随访情况(表 3)。

**3:** CCT文献的质量评价 Quality assessment of CCT studies

Included studies	Selection	Comparability	Outcome	Total
Fang ZM 2015^[8]^	4	2	1	7
Zhang XG 2015^[18]^	4	1	1	6
Liu YY 2015^[11]^	4	2	1	7
Qiao K 2013^[12]^	4	2	2	8
Wang CY 2015^[15]^	4	2	1	7

### 2.3 *Meta*分析结果

#### 2.3.1 术后住院时间

9项研究报道了术后住院时间，但1项研究^[12]^未提供标准差(SD)，故采用8项研究的数据。其中采用胸腔镜手术(video-assisted thoracoscopic surgery, VATS)的研究4篇^[8, 11, 16, 17]^，采用开胸手术的研究4篇^[9, 10, 15, 19]^，共754例患者，其中FTS组374例，CTS组380例。各研究之间存在异质性(*P* < 0.000, 01, *I*^2^=98%)，采用随机效应模型。*Meta*分析结果显示，与CTS组比，FTS组的术后住院时间明显减少，差异具有统计学意义(MD=-3.61, 95%CI: -5.05--2.16, *P* < 0.000, 01)(图 2)。按研究设计的类型进行亚组分析，仍发现在RCT和CCT的研究中FTS组均能减少术后住院时间(*P*_RCT_=0.002, *P*_CCT_ =0.004)(表 4)。

**2:**
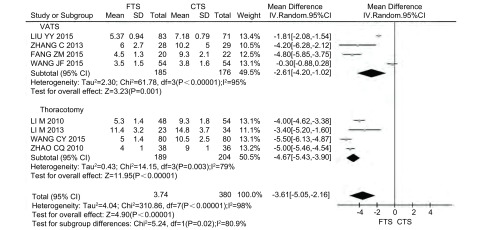
FTS组与CTS组术后住院时间的比较 Comparison of the length of postoperative hospital stay between FTS group and CTS group

**4:** 研究设计类型的亚组分析 Subgroup analysis of studies types

Item	No. ^a^	FTS/CTS	MD/OR (95%CI)	*P*_h_^b^	*I*^2^ (%)	*P*^c^
Postoperative hospital stay						
RCT	5	191/207	-3.35 (-5.49, -1.22)	< 0.000, 01	97	0.002
CCT	3	183/173	-4.02 (-6.78, -1.26)	< 0.000, 01	98	0.004
TOTAL	8	374/380	-3.61 (-5.05, -2.16)	< 0.000, 01	98	< 0.000, 01
Chest tube drainage						
RCT	5	186/198	-2.40 (-3.06, -1.73)	< 0.000, 01	87	< 0.000, 01
CCT	3	143/133	-2.97 (-3.62, -2.32)	0.003	83	< 0.000, 01
TOTAL	8	329/331	-2.62 (-3.07, -2.17)	< 0.000, 01	85	< 0.000, 01
Incidence of postoperative complications						
RCT	6	222/236	0.29 (0.16, 0.52)	0.09	47	< 0.000, 1
CCT	4	229/224	0.32 (0.14, 0.75)	0.05	62	0.009
TOTAL	10	451/460	0.30 (0.19, 0.47)	0.04	48	< 0.000, 01
Incidence of pulmonary infections						
RCT	6	222/236	0.35 (0.17, 0.71)	0.81	0	0.003
CCT	3	149/144	0.28 (0.09, 0.89)	0.59	0	0.030
TOTAL	9	371/380	0.33 (0.18, 0.60)	0.90	0	0.000, 3
Hospitalization costs						
RCT	3	91/100	-0.99 (-1.31, -0.68)	0.02	75	< 0.000, 01
CCT	1	20/22	-0.70 (-1.01, -0.39)	－	－	< 0.000, 01
TOTAL	4	111/122	-0.92 (-1.19, -0.65)	0.01	73	< 0.000, 01
^a^: No. of studies; ^b^: *P* value of Q test for heterogeneity; ^c^: *P* value of significance (including *P*_RCT_, *P*_CCT_); FTS: fast track surgery; CTS: conservative treatment surgery.						

#### 2.3.2 胸管留置时间

8项研究报道了胸管留置时间，其中采用VATS的研究6篇^[8, 11, 13, 16-18]^，采用开胸手术的研究2篇^[9, 20]^，共660例患者，其中FTS组329例，CTS组331例。各研究之间存在异质性(*P* < 0.000, 01, *I*^2^=85%)，采用随机效应模型。*Meta*分析结果显示，FTS组比CTS组的胸管留置时间明显减少，差异具有统计学意义(MD=-2.62, 95%CI: -3.07--2.17, *P* < 0.000, 01)(图 3)。亚组分析仍表明RCT和CCT的研究中FTS组均能明显减少胸管留置时间(*P*_RCT_ < 0.000, 01, *P*_CCT_ < 0.000, 01)(表 4)。

**3:**
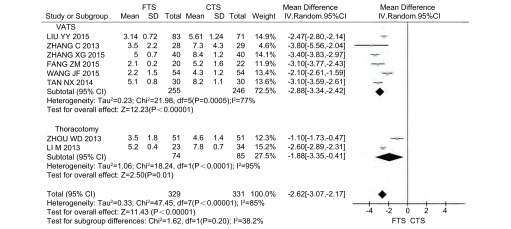
FTS组与CTS组胸管留置时间的比较 Comparison of chest tube drainage between FTS group and CTS group

#### 2.3.3 术后并发症发生率

术后并发症包括肺部感染、肺不张、心血管疾病、心律失常、切口感染、呼吸衰竭等。10项研究报道了术后并发症发生率，其中采用VATS的研究6篇^[8, 12, 13, 16-18]^，采用开胸手术的研究4篇^[9, 14, 15, 20]^，共911例患者，其中FTS组451例，CTS组460例。各研究之间存在异质性(*P*=0.04, *I*^2^=48%)，采用随机效应模型。*Meta*分析结果显示，与CTS组相比，FTS组能降低术后并发症发生率，差异具有统计学意义(OR=0.30, 95%CI: 0.19-0.47, *P* < 0.000, 01)(图 4)。按研究设计类型进行亚组分析同样表明在RCT和CCT的研究中FTS组仍能降低术后并发症发生率(*P*_RCT_ < 0.000, 1, *P*_CCT_=0.009)(表 4)。

**4:**
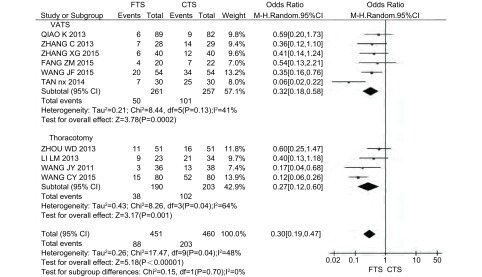
FTS组与CTS组术后并发症发生率的比较 Comparison of the incidence of postoperative complications between FTS group and CTS group

#### 2.3.4 术后肺部感染发生率

由于术后肺部感染与患者康复进程密切相关，因此将术后并发症中肺部感染发生率单独进行分析。9项研究报道了术后肺部感染发生率，其中采用VATS的研究6篇^[8, 12, 13, 16-18]^，采用开胸手术的研究3篇^[9, 14, 20]^，共751例患者，其中FTS组371例，CTS组380例。各研究之间无统计学异质性(*P*=0.90, *I*^2^=0%)，采用固定效应模型。Meta分析结果显示，与CTS组相比，FTS组能降低术后肺部感染发生率，差异具有统计学意义(OR=0.33, 95%CI: 0.18-0.60, *P*=0.000, 3)(图 5)。亚组分析得出同样结论，在RCT和CCT的研究中FTS组均能降低术后肺部感染发生率(*P*_RCT_=0.003, *P*_CCT_=0.03)(表 4)。

**5:**
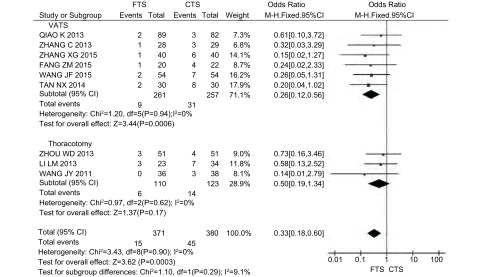
FTS组与CTS组术后肺部感染发生率的比较 Comparison of the incidence of pulmonary infections between FTS group and CTS group

#### 2.3.5 住院费用

4项研究报道了住院费用，其中采用VATS的研究2篇^[8, 13]^，采用传统开胸手术的研究2篇^[9, 19]^，共233例患者，其中FTS组111例，CTS组122例。各研究之间存在异质性(*P*=0.01, *I*^2^=73%)，采用随机效应模型。Meta分析结果显示，FTS组较CTS组缩减住院费用，差异具有统计学意义(MD=-0.92, 95%CI: -1.19--0.65, *P* < 0.000, 01)(图 6)。亚组分析同样表明FTS组较CTS组缩减住院费用(*P*_RCT_ < 0.000, 01, *P*_CCT_ < 0.000, 01)(表 4)。

**6:**
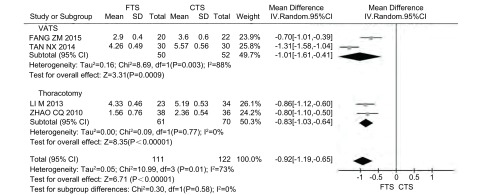
FTS组与CTS住院费用的比较 Comparison of the hospitalization costs between FTS group and CTS group

### 2.4 敏感性分析

基于上述的*meta*分析结果，对具有异质性的结果利用逐一剔除法进行敏感性分析。逐一剔除其中的每篇文献后重新计算MD或OR值，分析发现，每一次剔除均未对结果产生实质性影响。因此各分析对其纳入的文献不敏感，研究结果比较稳定。

## 3 讨论

本研究发现，与CTS相比，FTS应用于我国肺癌手术患者无论有无联合胸腔镜均有以下几个优势：①减少患者胸管留置时间；②降低肺部感染、肺不张、切口感染等术后并发症发病率；③减少术后住院时间；④减少住院费用。这有助于减轻患者痛苦和经济负担，提高病区床位周转率，节约医疗资源。

尽管如此，FTS却是有选择的在肺癌患者中使用，如具有肿瘤侵犯其他器官、心肺功能严重不全、合并严重心脑疾病等情况的患者则被排除在应用范围之内，FTS并未能让这些危重患者受益。随着我们对外科生理学认识的提高、医疗技术的进一步发展，FTS在不久的将来也许能安全的使用在这些危重患者上。而存在实施FTS途中不能耐受或依从性差等情况的患者，也会被排除在研究之外，这样可能会导致对FTS应用效果的评价偏高。因此，在实施FTS时，应根据手术方式和病情做适当的调整，对于肺癌Ⅲb期、Ⅳ期或手术耐受性较差的患者更应谨慎实施，以确保患者安全。

是否会给患者带来更高的术后并发症风险是实施及评价FTS的一个重点。本研究结果显示，实施FTS并未增加术后并发症发生率，反而使其有所降低。如术中加强保温措施，使患者体温维持在36 ℃左右^[21]^，可以避免低体温后复温时产生的应激损伤，有效预防术后心血管并发症的发生^[9, 13, 14, 16, 19, 22]^；控制术中、术后补液量在1, 000 mL-1, 500 mL以内有利于减少肺间质水肿引起肺部感染的发生率；通过术中肋间神经冰冻^[17, 19]^、术后自控式硬膜外镇痛泵镇痛术^[13, 14]^等方式减轻疼痛，减少因疼痛产生的应激反应，并有利于咳嗽咳痰和早期下床^[7]^，减少肺部感染和肺不张的发生率；术后24 h患者膀胱功能恢复后^[8-10, 14, 18]^甚至术后6 h^[7, 8, 13, 17]^即可拔出导尿管，有助于减少尿路感染。

与国外应用于肺癌的FTS措施相比，国内主要有两点不同：①胸管留置时间：其争议主要在于胸腔积液量小于多少时可拔管。国外有研究者^[23]^认为胸腔积液量 < 450 mL/d即可安全拔管；而我国研究者^[8, 12, 13, 24]^大多主张胸腔积液量 < 300 mL/d时拔管，还有研究者^[25]^认为肺叶切除术联合VATS可以不留置胸腔引流管。②术后下床时间：术后早期下床活动有利于减少血栓形成、肺部感染等并发症。国外有研究^[26]^表明肺叶切除术后4 h即可下床；而我国研究大多采取术后麻醉清醒后在床上坐起或半卧位活动四肢，术后第1天^[9, 10, 12, 13, 18]^或拔除胸管后^[8, 19]^下床活动。这可能由于国内外患者体能和文化的差异、医疗环境和水平的不同造成。

FTS应用于结直肠手术最为成功，其共识指南早在2005年就已发布，并于2013年又发布了最新正式指南^[27]^，而FTS应用于我国肺癌手术患者却还未形成共识，这在一定程度上会阻碍FTS有效实施。FTS的成功高度取决于多学科的协同治疗和患者的依从性^[28]^，因此麻醉师、医生、护士、理疗师、门诊医务人员、患者及其家属应团队协作，不断优化FTS措施，早日达成共识，充分发挥其优势，推广在临床的使用范围。

本研究同时纳入研究设计类型为RCT和CCT的文献，但通过对其进行亚组分析，发现这并未对结果造成干扰，RCT和CCT研究下FTS组相对于CTS组均表现出明显优势。本研究部分结果存在异质性，这可能由于各纳入研究之内及之间存在的差异。研究内的异质性主要由于各研究的样本含量不一致造成的。研究间的异质性主要是由于研究对象群体的不同，偏倚的控制方法等方面不一致造成的。

本次*meta*分析的所有纳入研究均符合纳入和排除标准，FTS组和CTS组均具有可比性，但仍存在以下局限性：①纳入研究均是中文文献，可能存在发表偏倚和语言偏倚；②5项CCT结果可能对研究结果造成过低或过高的评估；③各研究的FTS措施不尽相同，不同地区的医疗水平和理念也有差异，存在一定程度的实施偏倚。

综上所述，从现有研究来看，FTS应用于我国肺癌手术患者是安全有效的，但由于本研究所存在的局限性，今后仍需大样本、多中心的随机对照研究。此外，各研究之间的治疗方案还存在一定的差异，尚未形成统一、标准的方案，还需不断优化措施以早日达成共识，使更多的肺癌患者受益。
